# Using Objective, Real-Time Measures to Investigate the Effect of Actual Physical Activity on Affective States in Everyday Life Differentiating the Contexts of Working and Leisure Time in a Sample with Students

**DOI:** 10.3389/fpsyg.2012.00602

**Published:** 2013-01-21

**Authors:** Martina Kanning

**Affiliations:** ^1^Division I Sport and Health Science, Department of Sport and Exercise Science, University of StuttgartStuttgart, Germany

**Keywords:** ambulatory assessment, context of working and leisure time, physical activity and well-being, affective reactions, physical activity in every day life

## Abstract

Multiple studies suggest that physical activity causes positive affective reactions and reduces depressive mood. However, studies and interventions focused mostly on structured activity programs, but rarely on actual physical activity (aPA) in daily life. Furthermore, they seldom account for the context in which the aPA occur (e.g., work, leisure). Using a prospective, real-time assessment design (ambulatory assessment), we investigated the effects of aPA on affective states (valence, energetic arousal, calmness) in real-time during everyday life while controlling for the context. Eighty-seven undergraduates students (Age: *M* = 24.6; SD = 3.2, females: 54%) participated in this study. aPA was assessed through accelerometers during 24-h. Palmtop devices prompted subjects approximately every 45 min during a 14-h daytime period to assess their affective states and the context. We analyzed within- and between-person effects with hierarchical modeling (HLM 6.0). Multilevel analyses revealed that both aPA and context influenced subsequent affective states. The interaction of aPA and context did predict energetic arousal only. State levels of affects did not differ between men and women. For both men and women, aPA in everyday life has an effect on individual’s affective states. For valence and calmness, it seems to be independent of the context in which the aPA occur. For energetic arousal, men reported to have lower feelings of energy and women reported to have more feelings of energy during leisure time compared to working episodes.

## Introduction

Positive effects of regular, moderately intense physical activity on mental health has been presented in the literature several times, with small to medium effect-sizes (e.g., recent meta-analyses for positive and negative psychological states: Arent et al., 2000; Puetz et al., 2006; Reed and Ones, 2006; Reed and Buck, 2009). Independent of the impact of physical activity on mental health, to be physically active is a significant behavior to prevent non-communicable diseases (World Health Organization, 2002). Furthermore, positive affective reactions caused during and after a physical active episode will strengthen the maintenance of a regular active life style (Williams et al., 2008).

However, most of the studies that investigated the association between mental health and physical activity focused on structured activity programs only, but rarely on activities during everyday life. Physical activities during every day life include all possible activities in which people are ambulating, walking, do some gardening, or doing some kind of structured exercise for instance. Except of structured sport, those activities done in every day life are often processed automatically and they were performed spontaneously. We named those activities “actual physical activities” (aPA).

If not only structured activities but also activities that were done spontaneously in every day life have positive impacts on affective states, people may be able to regulate their mood in every day life. To encourage the potential effect of aPA on affect, it is important to know which moderators will have an important impact. A crucial moderator may be the context in which aPA takes place. Especially activities during daily routine are mostly embedded in different contexts, that is, the person is active at his or her working place, during leisure time or transport or he or she does some physically active household chores.

Up to now, it is unclear if aPA impacts affective states and whether this association depends on the context. It may be that the effect of aPA on affects is similar during working episodes or during leisure time, but evidence is missing, according to our knowledge. Information about the effects of aPA on affective states during daily routine within different contexts is worth to collect. It has often been shown that breaking times in sedentariness (Healy et al., 2008) or small amounts of moderate intense active episodes like Non-Exercise Activity Thermogenesis (NEAT Levine et al., 2005) have a substantial risk reducing impact on metabolic (e.g., diabetes type 2) and cardiovascular health (Wen et al., 2011).

Kanning and Schlicht (2010), as well as Schwerdtfeger et al. (2008, 2010) assessed affective states and aPA repeatedly during the day. Their results showed that affective states were significantly and positively associated with preceding aPA in every day life. However, the authors did not analyze any moderators that may influence the association in every day life, like the context in which subjects had been or sex differences.

To assess the associations between aPA, affective states, and the relevant context (working, leisure time, transport, chores) in real-time during every day life, we conducted an ambulatory assessment in which aPA was assessed by accelerometer and affective states and the context with electronic diaries.

We hypothesized that aPA in every day life as well as the context in which subjects had been have an impact on affective states. Especially, we were interested in the interaction between aPA and its context on affect. Therefore, we explored whether the context moderated the effect of aPA on affective states in everyday life. In addition, we analyzed sex differences. The meta-analyses about the effects of structured exercise on subjective well-being or affect did not report significant effect differences between men and women (e.g., Arent et al., 2000; Puetz et al., 2006; Reed and Ones, 2006; Reed and Buck, 2009). Thus, we hypothesized that the effect of aPA in every day life on affective states is not different between men and women.

## Materials and Methods

### Subjects

A convenience sample of 87 undergraduate students of sports science (47 females and 40 males) was recruited from a German university (*M* age = 24.6 years; SD = 3.2) in 2009. Before data recording, subjects were informed about the aim of our study. Following institutional ethical approval, subjects provided informed consent. Subjects received no monetary compensation for their participation.

### Ambulatory assessment procedure

Actual physical activity was measured continuously using a three-way accelerometer (varioport-e; Becker Meditech, Germany) for 24 h. The varioport-e was started and then attached to each subjects’s hip. Twenty-four hours later, subjects returned to the laboratory where data recording was stopped. Participants were allowed to take off the accelerometer when sleeping. Ratings for affect and context were assessed via electronic diaries (Palm, Tungsten E2). For this, the palmtop prompted subjects randomly about every 45 min during a defined 14-h daytime period (8:00 a.m. to 10:00 p.m.). If the person made no entry to the electronic diary, the palmtop beeped again 10 min later to remind the person to fill in the questionnaire. The assessment took always place on weekdays.

### Measures

#### Affective state

To assess momentary affective state we applied a short scale with six items based on the Multidimensional Mood Questionnaire (MDMQ; Steyer et al., 1997), which has been explicitly developed and evaluated for use in ambulatory assessment (Wilhelm and Schoebi, 2007). The scale contains six items measuring the basic affective states *valence* (unwell vs. well), *calmness* (relaxed vs. tense), and *energetic arousal* (tired vs. awake) by two bipolar items for each subscale. Wilhelm and Schoebi (2007) assessed homogeneity for the between-person level and for the within-person level. The level-specific reliability coefficient reached for the between-person level 0.92 for *valence* and 0.90 for *energetic arousal*, and *calmness*. The reliability coefficient for the within-person level reached 0.70 for *valence* and *calmness* and 0.77 for *energetic arousal*, resulting both in satisfying internal consistency. Subjects indicated on a six-point scale the extent to which they were experiencing different affective states. Answers were given by moving a slider from the left (“0,” i.e., “discontent”) to the right (“5,” i.e., “content”) end of the bipolar scale. Scores for each subscale were obtained by summing item scores resulting in a range from “0” (low value) to “10” (high value). We changed the Likert scale of the original version because we wanted to have a scale with an even number to force the sample to decide between the two poles.

#### Context

Subjects chose one out of the four following categories on their electronic diaries to define the context in which they had been: *work*, *transport*, *chores*, or *leisure time*. Subjects were prompted 831 times (52%) during leisure activities and 366 times (23%) during their work. For our sample working episodes could be student-related work or maybe job-related work if they have a part-time job. Because we achieved only a small number of episodes for transport (14%) and even fewer for chores (8%), we only account for episodes of leisure time and student-related work in the analyses. Therewith, the analyses did not account for 386 (25%) measurement points in which participants neither indicated leisure time nor working episode. Accordingly, we created a dummy-coded indicator variable named *Leisure/Work* (LW) with *leisure time* coded “1” and *work* coded “0.”

#### Actual physical activity

The varioport-e measured acceleration (defined as change in velocity over time) and describes the intensity, the rate of occurrence, and the duration of an actual physically active episode. Acceleration was measured in milligrams, than separated offline into AC and DC components by a FIR digital filter with a cut-off frequency set to 0.5 Hz. Raw signal, DC-values, and rectified AC-values were averaged across data points for each minute in the 24-h period. All offline analyses and artifact-checks were performed by the interactive software package “Freiburg Monitoring System” according to a published procedure (Myrtek, 2004). In order to analyze lagged within-subject relations, we aggregated the preceding 10 min of aPA before each entry into the electronic diary.

### Data analyses

We applied multilevel analyses using the statistical program HLM 6.0 (Raudenbush et al., 2004). Multilevel analyses were conducted separately for each affect subscale: *valence*, *energetic arousal*, and *calmness*. At first, we tested an unconditional model, where “*y*” is not modeled as a function of another variable at level-1 or level-2. Results from these three models revealed that the average levels of *valence*, *energetic arousal*, and *calmness* were *M* = 5.2, 4.4, and 5.1, respectively, which represented a daily affective state in the neutral range. The intra-class correlation was ρ*_I_* = 0.69 for *valence*, ρ*_I_* = 0.45 for *energetic arousal*, and ρ*_I_* = 0.65 for *calmness*.

In the following step, we entered consecutively the predictor variables aPA and LW and the interaction of these two predictors aPA *x* LW into our model. After level-1 models were finalized, we analyzed how significant the level-1 intercept and the level-1 predictors vary as a function of *sex*. The level-2 predictor sex was entered in all level-2 equations. Thus, we examined for each subscale the relationship between the level-1 intercept and sex (Eq. 2) and the cross-level interaction between level-1 slopes and sex (Eqs 3–5).

$$\begin{array}{llll} & \text{Level-}1:\;Y_{ti}=b_{0i}+b_{1i}{\left(\text{aPA}\right)}_{ti}+b_{2i}{\left(\text{LW}\right)}_{ti} & & \\ & \;\;\;\;+b_{3i}{\left(\text{aPA}x\text{LW}\right)}_{ti}+r_{ti} & & \text{(1)}\\ & \text{Level-}2:\;b_{0i}=\gamma_{00}\left(\text{sex}\right)+\mu_{0i} & & \text{(2)}\\ & \text{Level-}2:\;b_{li}=\gamma_{10}+\gamma_{11}\left(\text{sex}\right)+\mu_{1i} & & \text{(3)}\\ & \text{Level-}2:\;b_{2i}=\gamma_{20}+\gamma_{21}\left(\text{sex}\right)+\mu_{2i} & & \text{(4)}\\ & \text{Level-}2:\;b_{3i}=\gamma_{30}+\gamma_{31}\left(\text{sex}\right)+\mu_{3i} & & \text{(5)} \end{array}$$

Level-1 represents within-subject effects. Equation 1 represents the subjects’ response (subscript *_i_*) given on one out of the three affect subscales (*Y_ti_*) in any given diary entry (subscript *_t_*). *Y_ti_* is defined as the average intercept of one subscale across all subjects (*b*_0*i*_) and three level-1 predictors aPA (*b*_1*i*_aPA*_ti_*), context (*b*_2*i*_LW*_ti_*) and the interaction between aPA and context (*b*_3*i*_aPA*x*LW*_ti_*). These predictors are group mean centered where group is referring to a person (level-2). Intercept and slopes are conceived as randomly varying. The random effect for the level-1 model is given by *r_ti_*, *which* is assumed to be normally distributed with a mean of “0” and a variance of σ^2^.

Level-2 expresses between-subject effects. It includes the fixed effects, γ, as the average intercepts and slopes across all persons, the predictor *sex*, and the random effects, μ_0*i*,_ μ_1*i*,_ μ_2*i*_, and μ_3*i*_. The random effects are assumed to be multivariate and normally distributed, both with expected values of “0.”

Tests of the significance were conducted. The Alpha-level was defined as *p* < 0.05. Restricted maximum likelihood estimations were used for the multilevel analyses. Considering the nested structure of the model, effect size estimation was done with effective degrees of freedom. Formula 6 calculated the *N*_effective_ of the model (Snijders and Bosker, 2011):

(6) $$N_{\text{effective}}=\frac{Nn}{\left[1+\left(n-1\right)*\rho_{I}\right]}$$

*Nn* indicates the number of measurement points, *n* stands for the average measurement points per person and ρ_*I*_ represents the intra-class coefficient of the mood-subscale of interest. Effective degrees of freedom are analyzed with *N*_effective_ minus the number of predictors. We calculate effect size *r*, using *t*-values and effective degrees of freedom. According to Cohen (1992) an effect size *r* of 0.24 is in accordance *d* = 0.5 and represents a medium effect size.

## Results

Our sampling protocol generated a total of 1,583 data points belonging to 87 subjects resulting in approximately 19 e-diary entries per subject. All participants answered in any case to the six affects items, but during 39 assessments (2.5%) the participants didn’t answer to the item of the context. During the 10 min preceding the e-diary entry, subjects did show aPA of 84.4 mg/min on average (range: 0.4–994.4 mg/min). For comparison, jogging episodes reveal about 1,000 mg/min., walking episodes about 350 mg/min., and pure sitting episodes about 10 mg/min. The average and SD of aPA at person level ranged from 28 to 188 mg/min (SD = 24–308 mg/min).

Variance components are displayed in Table 1. Random error terms of some slopes were not significant in all models. They had to be fixed because the random and the fixed variability of that slopes cannot be reliably separated. For *valence* and *calmness*, aPA had to be fixed. LW had to be fixed for *energetic arousal*. Furthermore, the interaction had to be fixed in all models. This implies that these coefficients may vary but they do not vary randomly as a function of *sex*.

**Table 1:** **Variance components of between-person effects are presented for intercepts and slopes**.

Variance components between subjects	Variance estimate	SE	*c*^2^ (df)	*p*-Value
**MODEL 1: VALENCE**				
Intercept (μ_0*i*_)	4.34	2.08	1963.04 (59)	<0.001
aPA slope (μ_1*i*_)	Fixed			
LW slope (μ_2*i*_)	0.36	0.60	85.64 (59)	0.013
Interaction slope (μ_3*i*_)	Fixed			
Level-1 (*r_ti_*)	2.00	1.42		
**MODEL 2: ENERGETIC AROUSAL**				
Intercept (μ_0*i*_)	2.81	1.67	1059.84 (82)	<0.001
aPA slope (μ_1*i*_)	0.00004	0.006	174.28 (82)	<0.001
LW slope (μ_2*i*_)	Fixed			
Interaction slope (μ_3*i*_)	Fixed			
Level-1 (*r_ti_*)	3.28	1.81		
**MODEL 3: CALMNESS**				
Intercept (μ_0*i*_)	3.94	1.98	1854.14 (59)	<0.001
aPA slope (μ_1*i*_)	Fixed			
LW slope (μ_2*i*_)	0.56	0.75	104.47 (59)	<0.001
Interaction slope (μ_3*i*_)	Fixed			
Level-1 (*r_ti_*)	1.96	1.40		

As shown in Table 2, both the aPA and the dummy-coded indicator variable LW influenced affective states. aPA showed a positive effect on *valence* and *energetic arousal* and a negative effect on *calmness*. Therewith, the more our subjects were physically active, the more they subsequently reported feeling well (*valence*), full of energy (*energetic arousal*), and agitated [calmness (–)]. The effects were each large in effect-sizes *r* = 0.36, 0.52, and 0.29 standardized effects were 0.07, for 0.33 and −0.07 for *valence*, *energetic arousal*, and *calmness*, respectively. Thus if aPA increased by 1 unit, valence will increase and calmness will decrease by 0.07 and energetic arousal will increase by 0.33. Data analysis revealed a significant effect of LW on *valence* (positive), on *energetic arousal* (negative), and on *calmness* (positive). Compared to working episodes, subjects reported during leisure time feeling more well (valence), feeling a greater sense of calm (*calmness*), and having less energy [*energetic arousal* (–)]. The effects of LW were moderate to large with effect-sizes *r* = 0.18 (standardized effect = 0.05) for *valence*, *r* = 0.20 (standardized effect = −0.07) for *energetic arousal*, and *r* = 0.32 (standardized effect: 0.1) for *calmness*, respectively. The interaction between aPA and LW did not show any significant effect on any affective state with *p* = of 0.74, 0.74, 0.86 for *valence*, *energetic* *arousal*, and *calmness*, respectively.

**Table 2:** **Within-subject fixed effects are presented for intercepts and slopes activities**.

Within-subject fixed effects	Coefficient	SE	*t*-Value (df)	*p*-Value
**MODEL 1: VALENCE**				
Intercept (*b*_0*i*_)	5.30	0.23	23.94 (82)	<0.001
Sex	0.03	0.46	0.07 (82)	0.94
aPA slope (*b*_1*i*_)	0.002	0.0005	4.13 (1188)	<0.001
Sex	−0.0003	0.001	−0.35 (1188)	0.72
LW slope (*b*_2*i*_)	0.27	0.14	1.97 (82)	0.05
Sex	−0.42	0.28	−1.51 (82)	0.13
Interaction slope (*b*_3*i*_)	0.0005	0.002	0.34 (1188)	0.74
Sex	0.004	0.003	1.28 (1188)	0.20
**MODEL 2: ENERGETIC AROUSAL**				
Intercept (*b*_0*i*_)	4.26	0.19	22.65 (82)	<0.001
Sex	−0.35	0.39	−0.91 (82)	0.37
aPA slope (*b*_1*i*_)	0.008	0.001	8.39 (82)	<0.001
Sex	0.002	0.002	1.34 (82)	0.19
LW slope (*b*_2*i*_)	−0.39	0.15	−2.72 (1188)	0.007
Sex	0.15	0.29	0.52 (1188)	0.60
Interaction slope (*b*_3*i*_)	0.01	0.003	0.33 (1188)	0.74
Sex	0.01	0.005	2.63 (1188)	0.009
**MODEL 3: CALMNESS**				
Intercept (*b*_0*i*_)	5.20	0.22	23.56 (82)	<0.001
Sex	−0.04	0.44	−0.09 (82)	0.93
aPA slope (*b*_1*i*_)	−0.002	0.0005	−3.38 (1188)	0.001
Sex	0.0009	0.001	0.84 (1188)	0.40
LW slope (*b*_2*i*_)	0.57	0.15	3.83 (82)	<0.001
Sex	−0.55	0.30	−1.83 (82)	0.07
Interaction slope (*b*_3*i*_)	0.0002	0.002	0.18 (1188)	0.86
Sex	0.004	0.003	1.5 (1188)	0.13

To investigate sex differences we analyzed the effects of intercept and slopes of level-1 with the level-2 predictor *sex*. As shown in Table 2, *sex* is not significantly related to the main effects of the three subscales of affective states. But there is a significant effect of sex on the interaction term for the subscale *energetic arousal* (*r* = 0.21, standardized effect: −0.07). Because this finding based on a double interaction effect of a cross-level interaction between a level-2 predictor sex and an interaction term on level-1, we did sub-group analyses for both male and female sample to analyze between-person effects for this interaction. This sub-group analyses showed a significant interaction for both sex only for the subscale *energetic arousal* (for male: coefficient: −0.0078, *t*_(523)_ = −2.37, *p* = 0.02, *r* = 0.28, standardized effect = −0.08; for female: coefficient: 0.0074, *t*_(665)_ = 2.52, *p* = 0.01, *r* = 0.25, standardized effect = 0.09).

## Discussion

In our data set aPA as well as the context (working vs. leisure time) did significantly predict affective states. Our subjects reported feeling well, full of energy, but agitated the more they were physically active. The findings are mostly consistent with that of prior studies (e.g., Puetz et al., 2006; Reed and Ones, 2006). However, we revealed greater effect-sizes and that might be due to the fact that we assessed in real-time when the presumed effect actually occurs with objective methods. Kanning and Schlicht (2010) used also ambulatory assessment but they did not objectively assess aPA and they did not use electronic diaries.

In contrast to the study of Kanning and Schlicht, 2010 and for example Markowitz and Arent, 2010) our findings showed that activities in everyday life negatively affected *calmness*. We propose that these differing results are due to our ambulatory assessment. Our subjects did not expect when they were prompted and we had the chance to ask them while they were being physically active. Maybe the relaxing effects of physical activity appear only after some time delay when the acute physiological side-effects of physical activity have been vanished (cf. Kanning et al., 2012). Further studies are needed to analyze the association between physical activity and calmness with different time lags.

The context in which subjects were the 10 min before the e-diary prompt had also an impact on affective states. Compared to working episodes, subjects felt better and calmer during leisure time. This finding is viable because most often, leisure time represents activities aimed at enjoyment and social interaction. Those social objectives and states of feeling are associated with an enhanced subjective well-being (Argyle, 1999). Contrary to our expectations, *energetic arousal* was lower during leisure time in our data. Searching for an explanation, we found that several subjects chose the context leisure time while taking care of their hygiene or while eating (e.g., brushing one’s teeth, having a meal). During such episodes, a person may not feel full of energy but could still feel well and calm.

The interaction between aPA and the context did reveal a significant impact only on the subscale energetic arousal. To clarify this effect, we did sub-group analyses: The interaction predicted a negative impact on feelings of energy (energetic arousal) for men and a positive impact on feelings of energy for women. Therewith, men reported to have lower feelings of energy during leisure time compared to student-related working episodes. In contrast, women reported to have more feelings of energy during leisure time compared to student-related working episodes. Furthermore, aPA did seem to have an impact on valence and calmness, independent of the context of work or leisure in which the students were physically active (c.f. Bucksch and Schlicht, 2006).

The last finding was in line with the result of the *Scottish Health Survey* (Hamer et al., 2009). This periodic survey included data from 19,842 individuals. The study examined the association of different types of self reported physical activity (chores, walking, sports) and psychological distress. The regression models showed that all types of activity were independently associated with a lower risk of distress. However, physical activity was not assessed objectively and distress was not assessed in real-time. Teychenne et al. (2008) reviewed the association between self reported physical activity and depression. They compared the effect of leisure time activities with a combination of leisure time and domestic activities (e.g., chores). Leisure time activities alone did show a stronger association with the risk of depression than the combination of leisure time and domestic activities, leading to the conclusion that the volume, not the type of activity, was positively associated with a lower likelihood of depression.

In contrast to these findings were the results of a cross-sectional study of 1,919 adults (Asztalos et al., 2009). The authors analyzed associations between different types of PA (e.g., sports participation, chores – but no occupational activities) and stress. The main outcome was that only participation in sports and no other types of PA was inversely associated with stress. Compared to the study presented here, these studies analyzed negative psychological states. They did not focus on positive affects. Further research is needed to clarify if physical activity in everyday life within different contexts has different effects on positive and negative psychological states.

Furthermore, the state level of *valence*, *energetic arousal*, and *calmness* did not vary between men and women. This finding seems to be incompatible with the fact that e.g., depression is more prevalent in women than men (Centers for Disease Control and Prevention, 2010). However, Diener et al. (1999), reported in their literature survey that men and women were approximately equal in global happiness. When differences were found they often disappeared when other demographic variables were controlled. Fujita et al. (1991) stated that sex accounted for less than 1% of the variance in happiness but over 13% of the variance in the intensity of emotional experience. Women seem to be more open to intense emotional experiences and they express more positive emotions than do men (Nolen-Hoeksema and Rusting, 1999). Thus, women may balance their higher negative effect, resulting in levels of affect similar to those of men.

Strength of the present study is that we used prospective, objective, real-time approach (ambulatory assessment). In most other studies, physical activity was assessed through retrospective self-reports. However, systematic reviews (Prince et al., 2008) have shown that subjective measures of physical activity (e.g., questionnaire, diary) do only correlate low to moderate to real objective measures of physical activity (mostly accelerometry). Similar criticism has been raised regarding the retrospective assessment of affective states, as the recall of affects is often based on biased storage and recollection of memories (Ebner-Priemer and Trull, 2009). Ambulatory assessment methodology has the potential to resolve both problems by investigating self-reports, physiology, or behavior in (nearly) real-time in everyday life using objective methods.

Despite this methodological strength, a number of limitations in our research warrant discussion. First of all, subjects were all students of sports science. They were not only younger, more educated, and generally qualified for university admissions, but the sample had predominantly a special kind of working episodes. Students-related work may be not similar to the working context of the majority of the population. Second, to reduce the time and effort required of the subjects in answering the e-diary questions, we assessed the context of the aPA with a single item, covering four possible contexts (work, transport, chores, leisure time). For some situations (e.g., brushing teeth) this might lead to problems in classification. Third, further moderators of aPA and affective states were not assessed (e.g., weight status). Fourth, the monitoring period of our study was only 1 day. Therewith, it is not possible to analyze within-person variations of aPA and affect across different days. In addition we only used the preceding 10 min before each e-diary assessment for the time-lagged analysis. Future studies might investigate the association between affective state and aPA by an interactive sampling strategy that automatically recognizes inactive and active episodes and subsequently triggers e-diary assessments.

## Conclusion

The results of this study suggest that physically active episodes in a student’s daily life, which could spontaneous or structured and planned has an effect on individual’s affective states. The more male or female students were physically active, the more they felt well and full of energy. Surprisingly, they felt agitated, too. The findings for valence and calmness were independent of the context. So, there were no difference in feelings between the two conditions leisure time and working. Interestingly, the effect of the interaction differed between men and women for the subscale energetic arousal. Men reported to have lower and women reported to have more feelings of energy during leisure time compared to student-related working episodes. Further studies should analyze to what extent the context, in which aPA take place, had different effects on feelings of energy for men and for women.

## Conflict of Interest Statement

The author declares that the research was conducted in the absence of any commercial or financial relationships that could be construed as a potential conflict of interest.
